# What is the best biological parameter to predict erectile dysfunction in men aged >55 years with type 2 diabetes?

**DOI:** 10.1111/jdi.13089

**Published:** 2019-07-08

**Authors:** Sitraka A Raharinavalona, Nicolas Chevalier, Claude Gruel, André‐Christian N'toutoum, Fritz‐Line Vélayoudom Céphise

**Affiliations:** ^1^ Department of Endocrinology and Diabetology University Hospital of Guadeloupe Les Abymes Guadeloupe France; ^2^ Department of Endocrinology, Diabetology, Reproduction Hôpital de l'Archet Centre Hospitalier Universitaire de Nice Université Côte d'Azur Inserm UMR U1065/UNS Nice France; ^3^ L.A.M.I.A EA‐4540 University of Antilles Guadeloupe France

**Keywords:** Bioavailable testosterone, Erectile dysfunction, Total testosterone

## Abstract

To date, there is no evidence regarding the best biological marker to predict erectile dysfunction (ED) in men aged >55 years with type 2 diabetes. This prospective study included data from men aged >55 years with type 2 diabetes. ED was assessed by the International Index of Erectile Function 15‐item survey. Total testosterone (TT) levels and bioavailable testosterone were measured; the free testosterone index was calculated. Data from 155 men (aged 64 ± 7 years) were explored. The prevalence of ED and testosterone deficiency was 78.7% and 34.8%, respectively. After univariate analysis, TT and bioavailable testosterone were associated with ED (*P* = 0.01). After multivariate analysis, and adjustment for age, body mass index, tobacco, alcohol, duration of diabetes, TT, bioavailable testosterone, vitamin D and high‐sensitivity C‐reactive protein, we found that only high‐sensitivity C‐reactive protein was significantly predictive of ED. TT could predict ED, but it lacks specificity. We found a potential role of high‐sensitivity C‐reactive protein as a predictive marker of ED in this targeted population.

## Graphical abstract

Men with ED had lower levels of total testosterone and bioavailable testosterone compared to men without ED but the free testosterone index was not different. Surprisingly, the hsCRP was higher in men with ED compared to the others. The multivariate analysis found a new potential role of hsCRP as a predictive marker of ED in men with diabetes, aged >55 years.

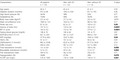

## Introduction

Erectile dysfunction (ED) is defined as an inability to have a sufficient erection for satisfactory sexual intercourse^1^. The frequency of ED is higher in men with type 2 diabetes compared with those without diabetes^2^. ED is usually associated with hypogonadism (HG) defined by low total testosterone (TT) levels^3^, ^4^. However, interpretation of TT is difficult in men aged >55 years, particularly in the case of type 2 diabetes. Some have proposed using bioavailable testosterone (BioT) or the free testosterone index (FTI) instead^5^, but no study could confirm their usefulness. We aimed to evaluate the relationship between ED and these three biological parameters in order to detect the best biological tool to predict ED in this population. Our findings could affect the clinical practice of physicians in the management of ED.

## Methods

We carried out a cross‐sectional study including all men aged >55 years with type 2 diabetes, after oral agreement, in the Department of Diabetology of the University Hospital of Guadeloupe. Patients with renal or prostate disease and cognitive disorders were excluded.

### Clinical data

ED was evaluated with the International Index of Erectile Function 15‐item (IIEF‐15) survey. The diagnosis of ED was confirmed when the IIEF‐15 score was <26^6^. Clinical parameters, including anthropometric data, were included.

### Biological assessments

Serum TT levels were measured using an electrochemiluminescence method. HG was defined as serum TT <10.4 nmol/L. According to the Vermeulen formula, BioT was obtained from a calculation using sex hormone‐binding globulin, albumin and TT^7^. Low BioT was defined by plasmatic levels <3.87 nmol/L. When the TT levels were between 8 and 10.4 nmol/L, the FTI was calculated. We also collected the levels of fasting blood glucose, glycated hemoglobin, lipids, 25(OH) vitamin D and high‐sensitivity C‐reactive protein (hs‐CRP).

### Statistical analysis

Data are presented as the mean ± standard deviation for continuous variables, and percentages (numbers) for categorical variables. Data were analyzed using the χ^2^‐test and *t*‐test, Pearson's and Spearman's correlation, and the odds ratio with a 95% confidence interval. Results were considered significant when *P* < 0.05 (two‐sided).

After approval by the local ethics committee, all men received a detailed note before they gave their oral informed consent. No men objected to the study.

In respect to the national legislation about data protection, a declaration of conformity has been made to the National Data Information and Freedom Commission.

## Results

We analyzed the data collected from 155 men aged >55 years with type 2 diabetes, included during 8 months in 2017. The characteristics of the population are summarized in Table 1. ED was self‐reported by 67.6% of the men, while its frequency was 78.7% using the IIEF‐15 survey. HG was found in 34.8% of patients.

**Table 1:** Characteristics of the study population

Characteristics	All patients *n* = 155	Men with ED *n* = 122	Men without ED *n* = 33	*P*‐value
Age (years)	64 **±** 7	63 ± 6	61 ± 5	0.18
Diabetes duration (months)	97.5 ± 84	104.2 ± 55.8	81.7 ± 79.5	0.36
High blood pressure (%)	26.5%	58%	53%	0.77
Dyslipidemia (%)	19.4%	40%	39.5%	0.39
Body mass index (kg/m²)	27.2 ± 4.3	27.7 ± 4.5	25.5 ± 3.5	0.079
Waist circumference (cm)	100.9 ± 13.3	102.4 ± 13.7	96.9 ± 10.6	0.17
Penile size (cm)	8.7 ± 2.6	8.2 ± 2.3	10.9 ± 2.5	**0.09**
Volume of testis (mL)	14.7 ± 5.1	13.9 ± 4.6	16.5 ± 6.1	**0.0001**
IIEF‐15 score	16.4 ± 9.2	13.3 ± 7.8	27.9 ± 1.2	**0.0001**
Fasting blood glucose (mg/dL)	148 ± 70	140 ± 64	141 ± 75	0.97
eGFR **(**mL/min/1.73 m^2^)	82.7 ± 20.4	90.1 ± 21.07	90.8 ± 13.4	0.90
AER (mg/day)	37.18 ± 66.5	32.67 ± 69.2	10.63 ± 10.65	**0.04**
HbA1c (%)	9.1 ± 3.1	10.1 ± 3.2	9.6 ± 3.6	0.59
25(0H)vitamin D (nmol/L)	86.5 ± 33.96	82.35 ± 33.33	97.13 ± 34.33	0.14
SHBG (nmol/L)	44.8 ± 24.9	40.7 ± 21.9	52.7 ± 38.9	0.12
Total testosterone (nmol/L)	13.1 ± 6.9	12.2 ± 7.4	18.8 ± 9.6	**0.006**
Bioavailable testosterone (nmol/L)	5.3 ± 2.3	4.9 ± 2.6	7.2 ± 2.7	**0.004**
Free testosterone index	34.4 ± 9.1	36.2 ± 8.3	33.6 ± 8.1	0.77
Hypogonadism (%)	34.8%	47.3%	20%	**0.078**
hs‐CRP use (mg/L)	5.76 ± 10.87	7.56 ± 12.06	1.69 ± 1.5	**0.001**

Data are presented as the mean ± standard deviation for continuous variables and percentages (numbers) for categorical variables. Results were considered significant when *P* < 0.05. The significant values are highlighted in bold. AER, albumin excretion rate; ED, erectile dysfunction; IIEF‐15, International Index of Erectile Function 15‐item version questionnaire; eGFR, estimated glomerular filtration rate obtained by Modification of Diet in Renal Disease Study method; HbA1c, glycated hemoglobin; SHBG, sex hormone‐binding globulin.

Comparison of clinical and biological parameters between men with or without ED is reported in Table 1. The sensitivity and specificity of TT for the diagnosis of ED were 89.66% and 29.27%, respectively.

BioT levels were low in 25.9% of patients. We observed a positive correlation between TT and BioT (*P* = 0.001), and an inverse correlation between TT levels and ED (*P* = 0.011). This inverse correlation was not observed for BioT and FTI.

After univariate analysis, TT and BioT were significantly associated with ED score (odds ratio [OR] 0.91, 95% confidence interval [CI] 0.84–0.98, *P* = 0.01 and OR 0.73, 95% CI 0.58–0.93, *P* = 0.01). No association was found between FTI and ED (*P* = 0.75). We carried out a multivariate analysis with adjustment for age, body mass index, tobacco, alcohol and duration of type 2 diabetes; we found that only TT was associated with ED (OR 0.92, 95% CI 0.84–1.00, *P* = 0.06). However, after additional adjustment for BioT, vitamin D and hs‐CRP, only hs‐CRP levels were associated with ED (OR 1.71, 95% CI 1.00–2.89, *P* = 0.046).

## Discussion

According to our knowledge, this is the first time that the three forms of testosterone evaluation are analyzed together as predictive factors of ED.

The subject of sexuality remains difficult to address, even in the field of health, which can explain why ED is not usually evaluated in men with type 2 diabetes, despite clear recommendations^1^, ^3^. According to a recent meta‐analysis, the prevalence of ED in diabetes patients was 52.5%^8^, lower than our findings of 78.7% using the IIEF‐15 score, targeting men aged >55 years. As the frequency of self‐reported ED was 67.6% in our cohort, we can assume that both physicians and patients themselves underestimated ED.

Although the IIEF‐15 is a simple and reliable tool for ED diagnosis, it remains difficult to determine the pathophysiological origin of ED. ED observed in type 2 diabetes can result from both neuropathy and distal arteriopathy^9^, ^10^. However, diabetes itself is associated with HG defined by low TT levels. Furthermore, it is well known that TT decreases with age, particularly after the age of 55 years and, finally, its measurement is only recommended in the case of symptoms^3^.

In the present cohort, we observed 34.8% of HG, defined as a serum level of TT under 10.4 nmol/L. Our levels of TT were closer to those described in previous studies^10^, ^11^. However, the frequency of HG was noticeably higher^11^. Considering the ED statute as the main outcome, we reported a good sensitivity, but a very low specificity of TT in our population, which highlights the lack of clinical usefulness of this biomarker.

The use of other markers of HG, such as BioT or FTI, has so far proposed been, but has not been confirmed yet. Furthermore, in a recent study carried out on a general population, independently of TT, higher sex hormone‐binding globulin was associated with either subjective or objective androgen deficiency features^12^. This remains to be confirmed in patients with type 2 diabetes.

The significant correlation between TT and BioT is relevant, but both parameters are not associated with ED in the same way. We confirmed here that FTI is not useful.

We highlighted the role of hs‐CRP as a possible good predictive marker of ED. The role of hs‐CRP on metabolic syndrome, associated or not with low TT, is well known, particularly in elderly healthy men aged >75 years, but studies targeting men with type 2 diabetes are lacking^13^. hs‐CRP is clearly known as a biomarker of the cardiovascular risk of people with type 2 diabetes or polycystic ovary syndrome. The predictive role of hs‐CRP for ED was previously described in obese people without type 2 diabetes 14. However, to our knowledge, it is the first time that its predictive role for ED in men aged >55 years with type 2 diabetes has been described and compared with TT, BioT and FTI. It has been previously reported that hs‐CRP levels were associated with a moderate‐to‐high cardiovascular risk in non‐diabetic men with moderate‐to‐severe ED^15^. Another study reported that hs‐CRP levels were higher in diabetes patients with ED compared with those without ED, but the present study did not target the specific population of men aged >55 years, and included younger men without measurement of the sex steroids levels^16^.

The impact of the present finding is very important, as a previous study reported that ED could be the first clinical sign of endothelial dysfunction and a clinical marker of cardiovascular diseases. Finally, our results could change the clinical management of men with diabetes, particularly for the screening of cardiovascular risk factors^17^.

## Conclusion

BioT or FTI are not good markers to evaluate ED in men aged >55 years with type 2 diabetes. TT lacks specificity in this studied population. However, unexpectedly, we found that hs‐CRP could have a potential role as a predictive marker of ED.

## Disclosure

The authors declare no conflict of interest.
